# Dietary fat and carbohydrate have different effects on body weight, energy expenditure, glucose homeostasis and behaviour in adult cats fed to energy requirement

**DOI:** 10.1017/jns.2014.60

**Published:** 2015-01-22

**Authors:** Margaret A. Gooding, Jim L. Atkinson, Ian J. H. Duncan, Lee Niel, Anna K. Shoveller

**Affiliations:** 1 University of Guelph, Animal and Poultry Science, Guelph, Ontario, Canada N1G 2W1; 2 Procter and Gamble Pet Care, Mason, OH 45040, USA; 3 Ontario Veterinary College, Population Medicine, Guelph, Ontario, Canada N1G 2W1

**Keywords:** Indirect calorimetry, Energy expenditure, Activity, Play, Cognition, Macronutrients, Insulin, BMC, bone mineral content, BW, body weight, DXA, dual-energy X-ray absorptiometry, EE, energy expenditure, G:I, glucose:insulin, HC, high carbohydrate, HF, high fat, LBM, lean body mass, ME, metabolisable energy, RQ, respiratory quotient

## Abstract

The effects of dietary carbohydrate and fat on feline health are not well understood. The effects of feeding diets moderately high in fat (HF; *n* 10; 30 % fat, 26 % carbohydrate as fed) or carbohydrate (HC; *n* 10; 11 % fat, 47 % carbohydrate), for 84 d, were investigated in healthy, adult cats (3·5 (sd 0·5) years). Data on indirect calorimetry, blood biomarkers, activity, play and cognition were collected at baseline, and at intervals throughout the study. Body composition was measured by dual-energy X-ray absorptiometry at baseline and on day 85. There were no significant main effects of diet on body weight and composition. When data were analysed over study day within diet, cats fed HF diets experienced a significant increase in body fat (*P* = 0·001) and body weight (*P* = 0·043) in contrast to cats consuming the HC diet that experienced no change in body fat or body weight (*P* = 0·762) throughout the study. Overall, energy expenditure was similar between diets (*P* = 0·356 (fasted), *P* = 0·086 (postprandial)) and respiratory quotient declined with exposure to the HF diet and increased with exposure to the HC diet (*P* < 0·001; fasted and postprandial). There was no difference in insulin sensitivity as an overall effect of diet (*P* = 0·266). Activity declined from baseline with exposure to both diets (HC: *P* = 0·002; HF: *P* = 0·01) but was not different between diets (*P* = 0·247). There was no effect of diet on play (*P* = 0·387) and cats consuming either the HF or HC diet did not successfully learn the cognitive test. Overall, cats adapt to dietary macronutrient content, and the implications of feeding HC and HF diets on risk for adiposity as driven by metabolic and behavioural mechanisms are discussed.

Approximately 50 % of cats (*Felis catus)* are overweight or obese in the USA^(^^1^^)^. Macronutrient distribution in the diet, namely diets high in fat or carbohydrate, as a percentage of energy, have been identified as risk factors for weight gain^(^^2^^,^^3^^)^ and remains a controversial area. For domestic cats, it remains unclear as to whether a high-carbohydrate (HC) or high-fat (HF) diet poses the greatest risk for the development of obesity and diabetes as both have been shown to contribute to adiposity and perturbations in glucose and insulin handling^(^^4^^–^^6^^)^. Further, metabolic effects of HF and HC diets, as observed in human subjects and rodent species, may be compounded by behavioural impacts that contribute to lethargy, impaired cognitive skills and reduced satiety, via specific brain responses, all factors that may increase the risk for weight gain^(^^7^^–^^9^^)^.

The objectives of the present investigation were to examine the metabolic and behavioural effects of feeding cats HF or HC diets for 84 d. We hypothesised that feeding a HC diet would lead to outcomes related to insulin insensitivity, but within the time frame of the present study, no improvements in behavioural outcomes.

## Materials and methods

All procedures were reviewed and approved by the Institutional Animal Care and Use Committee at Procter & Gamble Pet Care.

### Animals and housing

A group of twenty cats of similar age (3·5 (sd 0·5) years), ten female and ten males, were provided by the Pet Health and Nutrition Center at Procter and Gamble Pet Care, Lewisburg, OH, USA. Veterinarian evaluation indicated that all cats entered the study healthy. Outside of calorimetry collection, cats were housed in a free-living group environment. On indirect calorimetry days, cats were temporarily housed in respiration calorimetry chambers (Qubit Systems^®^) made of Plexiglas and measuring 53·3 × 53·3 × 76·2 cm. Cats had been previously acclimatised to chambers over an 11-week period of increasing exposure^(^^10^^)^ and routinely exposed to maintain acclimatisation.

### Diets

HF and HC diets were formulated similarly on an ingredient and nutrient content basis as a percentage of energy, had similar dietary protein:metabolisable energy (ME) ratios (Table 1), and were fed to equal ME as a function of body weight (BW) (females = 189 kJ ME/kg BW per d; males = 209 kJ ME/kg BW per d)^(^^11^^)^. Feeding in this manner avoids confounding effects due to differences in absolute protein and energy intake^(^^12^^)^. The dietary nutrient content of each diet was determined before the start of the study using duplicates and AOAC^(^^13^^)^ procedures for DM (934·01), CP (990·03), acid-hydrolysed fat (954·02), fibre (962·09), starch (979·10), ash (942·05), P (964·06) and Ca (968·08). Dietary gross energy was determined using an automated bomb calorimeter (IKA C-2000; IKA Works, Inc.). Cats were fed individually at 07·00 hours and allowed 60 min to consume the meal. Remaining feed was collected and weighed and daily energy intakes recorded for each cat. The washout diet fed during baseline collections was Iams^®^ Original Chicken diet.

**Table 1.** Ingredient composition (g/kg) and analysed nutrient contents (% of metabolisable energy; ME) of the high-fat (HF) and high-carbohydrate (HC) diets (as-fed)

Diet…	HF	HC
Ingredient (g/kg)		
Chicken by-product meal	440·7	291·4
Maize, yellow	179·9	277·6
Chicken fat	182·1	20·0
Maize grits	–	228·8
Sorghum grain	60·0	92·5
Beet pulp	24·7	25·6
Fish oil	23·4	3·6
Chicken flavour	19·0	19·0
Dicalcium phosphate	14·7	19·6
Dried egg	9·9	10·2
Sodium bisulfate	8·0	8·0
Potassium chloride	6·7	6·3
Mineral premix*	5·0	5·0
Yeast, brewers	4·9	5·1
Calcium carbonate	4·2	5·3
Choline chloride	3·9	3·1
dl-Methionine†	0	0·9
Vitamin premix‡	1·6	1·0
Analysed nutrient content (%)		
Moisture	8·0	8·0
Protein	34·0	30·0
Fat	30·0	11·0
Crude fibre	1·0	1·5
Ash	5·0	6·0
NFE§	25·8	47·1
Calculated ME (kJ/kg)||	19 907	15 631
Protein (g/kg):ME (kJ/kg)¶	0·002	0·002

NFE, N-free extract; ppm, parts per million.

* Mineral premix contained: 40·4 % K, 38·1 % Cl; 3500 ppm Cu; 16 120 ppm Mn; 60 000 Zn; 420 ppm I; 150 ppm Co.

† Diets were formulated to contain 1·150 g dl-methionine/kg.

‡ Vitamin premix contained: 36 300 kIU/kg vitamin A; 1 725 000 IU/kg vitamin D_3_; 148 650 IU/kg vitamin E; 22 575 ppm thiamine; 89 130 ppm niacin; 19 200 ppm pyridoxine; 25 935 ppm pantothenic acid; 2430 ppm folic acid; 189 ppm vitamin B_12_; 5520 ppm inositol; 54 000 ppm vitamin C; 540 ppm biotin; 5940 ppm riboflavin.

§ NFE = 100 – (crude protein + crude fat + crude fibre + moisture + ash)^(^^10^^)^.

|| ME was calculated from guaranteed analysis and the modified Atwater equation (ME (kJ/kg) = (14·6 × kg NFE) + (35·6 × kg fat) + (14·6 × kg protein))^(^^11^^)^.

¶ Diets were formulated to contain the same protein:energy ratio.

### Experimental design

For 3 weeks, all cats were fed the washout diet and subjected to baseline measurements. After baseline collections cats were blocked based on body condition and sex and allocated to either a HF or HC dietary treatment for 84 d. On days –7, 35 and 76, cats were subjected to 22 h indirect calorimetry measurements and upon removal from the chambers blood (3·5 ml) was sampled. On days –14, 28 and 70, activity was measured, on days –20 and 64, play motivation was measured, and on days –1, 42 and 84, cognitive function was measured. BW was measured weekly and feed intake daily. Body composition was measured using dual-energy X-ray absorptiometry (DXA) on day –21 of the washout diet and on day 85 for all cats.

### Body composition

For DXA, animals were anaesthetised with an intramuscular injection of Dexdomitor^®^ (Pfizer Corp.) in combination with Hydromorphone^®^ (Baxter Healthcare Corp.). Three DXA scans were completed for each cat and data averaged (Hologic Inc.). Once the scans were completed, an intramuscular injection of a reversal agent Antisedan^®^ (Pfizer Corp.) was administered.

### Indirect calorimetry and blood metabolites

To assess the effects of diet, indirect calorimetry, a validated technique for the measurement of resting energy expenditure (EE) in cats^(^^14^^)^, was used to measure respiratory gas exchange to calculate EE and respiratory quotient (RQ)^(^^15^^)^. Fasted blood metabolites measured included: glucose and insulin. Indirect calorimetry and blood sampling procedures have been described previously^(^^16^^)^.

### Behavioural assessments

Voluntary activity was measured using Actical^®^ activity monitors (Mini Mitter) that were worn parallel to the ribs and attached via a harness for 24 h. The Actical^®^ software analysed and converted the data into arbitrary numbers referred to as activity counts per designated time period (15 s).

Play motivation was measured 6 h post-feeding using an obstruction test^(^^17^^)^. Two boxes (width 100 cm, length 100 cm, height 75 cm; Queen City Polymers), one start box and one goal box, were connected via a swing door (width 23 cm, height 18 cm) made of 1·9 cm (1/16 feet) Plexiglas which is similar to the doors that are in the group housing to access outside. To assess play motivation, the swing door was made progressively more difficult to open through the addition of weights (maximum 600 g). When the cat pushed at the weighted door with sufficient force the cat could pass underneath the door to enter the goal box where interaction with a toy mouse was permitted for 30 s. The maximum door weight that each cat would push to enter the goal box was measured.

A T-maze (stem: length 2·13 m (7 feet), width 0·46 m (1·5 feet), height 0·46 m (1·5 feet); arm: length 0·99 m (3·25 feet), width 0·46 m (1·5 feet), height 0·46 m (1·5 feet)) was used to measure cognitive function 6 h post-feeding. All cats were acclimatised to the T-maze and associated testing before the study. A spatial cue (a circle and letter ‘x’) was randomly assigned as a positive (rewarded) and negative (non-rewarded) cue for each cat and balanced for diet. Ten trials per day were used to measure the number of correct arm entries. Both arms were baited with 1 g of food to ensure that olfactory cues did not influence performance; however, food was only accessible to cats if they entered the correct arm containing that cat's positive (rewarded) cue.

### Statistical analysis

All statistical analyses were performed using SAS (version 9.1; SAS Institute Inc.)^(^^18^^)^. Mixed-effect models were fitted using the PROC MIXED function and the dependent variables were analysed using repeated measures where the fixed effects were diet and day and the random variable was cat. Denominator df were calculated using the Kenward–Rogers approximation. Repeated measures were analysed using the covariance matrix that had the smallest Akaike information criterion value and multiple comparisons were made using the Tukey–Kramer method. Fixed (main) effects of diet, day and diet × day interactions are reported; in addition, all significant or trending effects of diet × day for differences of least-squares means are discussed. Differences were considered significant at *P* < 0·05. All data are represented as least squares means with their pooled standard errors.

## Results and discussion

### Body weight and composition

There were no significant main effects of diet, day and diet × day on BW and lean body mass (LBM); however, there was an overall significant effect of day on total body fat (*P* > 0·05), with no significant main effect of diet and diet × day. When specific effects within diet across day were reviewed, an increase in BW (*P* = 0·043; day –21 (4·76 (sem 0·39) kg) *v.* day 85 (4·9 (sem 0·44) kg)) occurred for cats fed the HF diet, but not the HC diet (*P* = 0·762; day –21 (4·91 (sem 0·39) kg) *v.* day 85 (4·89 (sem 0·44) kg)). This was due to an increase in body fat (*P* = 0·001; day –21 (0·74 (sem 0·22) kg) *v.* day 85 (1·00 (sem 0·23) kg)) and trend towards a decline in LBM and bone mineral content (BMC) (LBM + BMC) (*P*= 0·071; day –21 (4·01 (sem 0·2) kg) *v.* day 85 (3·91 (sem 0·2) kg)) in the cats fed the HF diet. As ME (calculated) intake did not differ over day (*P* > 0·05) or between diet (*P* = 0·821; HC (171·46 (sem 7·61) kJ/kg BW per d) *v.* HF (168·95 (sem 7·61) kJ/kg BW per d)), the changes in body composition were caused by differences in macronutrient composition as a main effect of diet only (*P* < 0·001; fat (69·04 (sem 4·1) kJ/kg^0·67^ per d for HC *v.* 161·08 (sem 4·1) kJ/kg^0·67^ per d for HF) and carbohydrate (144·35 (sem 4·1) kJ/kg^0·67^ per d for HC *v.* 62·76 (sem 4·1) kJ/kg^0·67^ per d for HF)). The present study agrees with previous results where cats had a greater risk for weight gain when fed HF *v.* HC diets either *ad libitum*^(^^5^^,^^19^^)^ or to energy requirements^(^^4^^,^^6^^)^. In addition, these results further support the hypothesis that using calculated values of ME systemically underestimates the true ME content of commercially available HF diets for cats^(^^20^^)^; therefore, it is unclear as to whether the increase in BW and body fat with HF dietary feeding was driven by fat or energy intake or both. Furthermore, the trend in a reduction in LBM + BMC require further investigation as the mechanism for a loss in structural protein is surprising and may suggest that, compositionally, more protein as a function of energy content should be considered.

### Macronutrient metabolism

There was a significant main effect of diet and diet × day (*P* < 0·05), with no significant effect of day (*P* = 0·679), on fasted RQ. For cats consuming the HF diet, postprandial RQ increased from day 35 to day 76 when the effect of day was compared within dietary treatments (*P* = 0·022; Table 2); as such, there was a significant main effect of diet, day and diet × day for postprandial RQ (*P* < 0·05). Fasted EE was not different between diets (*P* = 0·356) and there was no main effect of diet × day (*P* = 0·697); however, there was a significant main effect of day (*P* = 0·001) which can be explained by the observed transient increase in fasted EE on day 35 (Table 2), an effect hypothesised to be due to an external environmental influence or as a function of dietary adaptation. There was a trend towards a main effect of diet on postprandial EE (*P* = 0·086) with a significant main effect of day (*P* = 0·013) and no effect of diet × day (*P* = 0·588). While postprandial EE was similar over the diet treatment period for cats fed the HC diet, postprandial EE decreased over the treatment period for cats fed the HF diet (*P* < 0·05; Table 2). Overall, with the HF diets the lower fasted and postprandial RQ suggest a partial increase in lipid use for energy as a proportion of all macronutrients; however, the decline in postprandial EE with increased length of exposure to the diet may have resulted in a positive energy balance and explain the increase in body fat. The decrease in EE is probably compounded by the tendency for LBM to decrease in cats fed HF diets since LBM is the major predictor of non-activity-related EE. A low heat increment of feeding did not appear to influence EE since the decline in EE was only observed after long-term exposure (day 76). Thus, the decline in EE may have been due to the diversion of lipids away from oxidation towards lipogenesis as we observed a concurrent increase in BW and fat with an increase in RQ with long-term exposure.

**Table 2.** Respiratory quotient (RQ), energy expenditure (EE) and blood metabolites in cats after exposure to high-fat (HF) and high-carbohydrate (HC) diets † (Least-square means (LSM) with their pooled standard errors; *n* 10)

		HC (*n* 10)	HF (*n* 10)			Main effects‡		
	Day	LSM	LSM	sem (pooled)	*P*_diet(day)_	*P*_diet_	*P*_day_	*P*_diet×day_
RQ fasted for 24 h	–7	0·791^b^	0·791^a^	0·01	1·00	0·001	0·679	0·003
	35	0·814^a^	0·763*^b^	0·01	<0·0001			
	76	0·814^a^	0·755*^b^	0·01	<0·0001			
RQ postprandial average	−7	0·834^b^	0·832^a^	0·002	0·724	<0·001	0·001	<0·001
	35	0·860^a^	0·774*^c^	0·004	<0·0001			
	76	0·863^a^	0·791*^b^	0·007	<0·0001			
EE fasted for 24 h (kJ/kg^0·67^ per d)§	–7	295·8^b^	277·8^b^	15·5	0·546	0·356	0·001	0·697
	35	319·7^a^	303·8^a^	17·2	0·644			
	76	310·9^a,b^	284·1^b^	15·1	0·497			
EE postprandial average (kJ/kg^0·67^ per d)§	–7	284·1	297·1^a^	6·3	0·178	0·086	0·013	0·588
	35	285·8	302·1^a^	6·3	0·079			
	76	273·6	279·1^b^	5·0	0·467			
Insulin (ng/l)	–6	158·4	163·8^a^	19·1	0·843	0·215	0·113	0·067
	36	145·1	130·6^b^	17·5	0·567			
	77	245·7	113·2^a,b^	57·0	0·118			
Glucose (mg/dl)‖	–6	86·4	86·8^b^	1·5	0·852	0·060	0·160	0·029
	36	83·9	93·0*^a^	1·4	0·0002			
	77	89·9	92·5^a,b^	3·5	0·604			
G:I	–6	0·61	0·61^b^	0·1	0·957	0·266	0·001	0·021
	36	0·62	0·84^a^	0·1	0·068			
	77	0·73	0·90^a^	0·1	0·325			

G:I, glucose:insulin.

^a,b,c^ Mean values in a column with unlike superscript letters were significantly different among day within diet (*P* < 0·05).

* Mean value was significantly different from that for the HC diet (*P* < 0·05; difference due to diet effects within day).

† *P* value refers to the ANOVA for diet within day effect and main effects of diet, day and diet × day.

‡ Main effects of diet, day and diet × day (*P* < 0·05).

§ Postprandial RQ and EE averages were calculated over 20 h post-feeding, with measures occurring at 30-min intervals.

‖ To convert mg/dl to mmol/l, multiply by 0·0555.

### Blood metabolites

There was a trend towards a significant main effect of diet on plasma glucose (*P* = 0·060), no effect of day (*P* = 0·160) and a significant interaction effect of diet × day (*P* = 0·029) on plasma glucose. Main effects were probably driven by the increase in glucose concentrations over day (*P* < 0·05; Table 2) for cats fed the HF diet and the diet effect observed on day 36 as cats fed the HC diet had lower glucose concentrations than cats fed the HF diet (*P* = 0·001). Though diets were fed to an equivalent protein:ME ratio amino acids may have been transiently utilised for gluconeogenesis resulting in greater serum glucose concentrations^(^^21^^)^ during diet adaptation^(^^16^^)^. There was no significant main effect of diet or day on insulin (*P* > 0·05); however, there was a trend towards an effect of diet × day interaction (*P* = 0·067). This trend may have been driven by the transient decline in insulin from baseline in cats fed the HF diet (*P* < 0·05; Table 2). There was no significant effect of diet on glucose:insulin (G:I) (*P* = 0·266). There was a significant main effect of day and diet × day on G:I (*P* < 0·05). When G:I was analysed across day within diet treatment there was a trend towards a higher ratio with HF feeding on day 36 (*P* = 0·068; Table 2). With the consumption of the HF diet less insulin was required to normalise plasma glucose; therefore, insulin sensitivity may have been improved for cats fed the HF *v.* HC diet. However, conclusions warrant further investigation as G:I ratio provides only a gross representation of insulin sensitivity and the effects of diet on G:I were not significant and only transient for cats consuming the HF diet, suggesting dietary adaptation.

### Physical activity

There were no significant main effects of diet or diet × day on physical activity (*P* > 0·05); however, there was a significant effect of day on voluntary activity (*P* = 0·001). Effects were probably driven by the decline in activity from baseline with exposure to both the HC (*P* = 0·002; slope = –1·44) and HF (*P* = 0·01; slope = –1·32) dietary treatments. A decline in voluntary physical activity is not surprising, as both diets high in carbohydrate and fat content have been shown to influence mood by reducing energy levels and alertness^(^^22^^,^^23^^)^; for instance, with HC dietary feeding the effects on glucose and insulin metabolism can influence the circulation of tryptophan, serotonin and the expression of brain noradrenaline transporters, decreasing the release of adrenaline that can make an impact on mood and energy^(^^8^^,^^24^^)^. Alternatively, HF diets, via an increase in release of cholecystokinin, a peptide hormone implicated in the mediation of postprandial sleepiness^(^^25^^,^^26^^)^, has been shown to promote greater feelings of lethargy^(^^27^^)^. Overall, activity declined in both dietary treatments and differences between diets may not have been observed because the cats were relatively weight stable.

### Play behaviour

There were no significant main effects of diet, day or diet × day on play motivation (*P* > 0·05). Maximum door weights on day 64 were 370 (sem 86) g and 410 (sem 86) g, for the HF and HC diet, respectively (*P* = 0·387). In the domestic cat, play motivation is influenced by, but not exclusive to, hunger^(^^28^^)^. Since diets high in fat and carbohydrates appear to have differing, but inconclusive, effects on satiety^(^^7^^)^, we may be able to hypothesise that the fat:carbohydrate ratio does not significantly influence satiety in the cat when play motivation is used as an indirect indicator of satiety or energy sensing^(^^29^^)^. However, measuring *ad libitum* intake and satiety hormones, in a study where energy intake is not controlled, is likely to provide a more direct and valid measure of the effect of macronutrient content on satiety.

### Cognition

Overall, there were no significant main effects of diet, day and diet × day on cognitive performance (*P* > 0·05). As groups were different at baseline, change from baseline was analysed and a significant main effect of diet (*P* = 0·041) on change in T-maze performance (HC = +0·85 *v.* HF = –0·85) was observed. However, the test was not successful as cats did not learn the task (as learning was defined as a mean score >70 %); thus, conclusions are only speculative. It may be hypothesised that the trend for improvement in performance with HC feeding was driven by certain cognitive functions that are sensitive to short-term variations in glucose availability; however, this conclusion warrants further investigation^(^^9^^,^^30^^)^. While cats consuming the HF diet had higher blood glucose levels after a 24 h fast, the blood glucose levels at the time of testing, 6 h after feeding, were probably higher in the cats consuming the HC diet^(^^19^^)^.

### Conclusion

In conclusion, cats are capable of adapting energy metabolism to different macronutrient intakes; however, after an 84 d feeding the diet higher in dietary fat presented a greater risk for the development of adiposity and associated metabolic effects if fed to a calculated ME allowance. It is unclear if effects are driven by total fat content of the diet or differences in energy intake, as it is understood that using calculated ME for diets high in fat may lead to an underestimation of energy density of the diet and ultimately contribute to overfeeding and increased BW and fat. Conversely, the consumption of a HC diet had minimal effects on energy metabolism and behaviour.
